# [Retracted] Chronic oxymatrine treatment induces resistance and epithelial‑mesenchymal transition through targeting the long non‑coding RNA MALAT1 in colorectal cancer cells

**DOI:** 10.3892/or.2024.8732

**Published:** 2024-04-09

**Authors:** Yibai Xiong, Jun Wang, Huirong Zhu, Lingshuang Liu, Yi Jiang

Oncol Rep 39: 967–976, 2018; DOI: 10.3892/or.2018.6204

Following the publication of the above article, a concerned reader drew to the Editor's attention that certain of the cell migration and invasion assay data featured in Figs. 2B, 5C, 6B and C were strikingly similar to data appearing in different form in other articles written by different authors at different research institutes that had either already been submitted elsewhere prior to the submission of this paper to *Oncology Reports*, or were under consideration for publication at around the same time (one of which has been retracted).

In view of the fact that certain of these data had already apparently been submitted for publication prior to the submission of this article to *Oncology Reports*, the Editor has decided that this paper should be retracted from the Journal. After having been in contact with the authors, they agreed with the decision to retract the paper. The Editor apologizes to the readership for any inconvenience caused.

